# Evolution of Disease Defense Genes and Their Regulators in Plants

**DOI:** 10.3390/ijms20020335

**Published:** 2019-01-15

**Authors:** Rongzhi Zhang, Fengya Zheng, Shugen Wei, Shujuan Zhang, Genying Li, Peijian Cao, Shancen Zhao

**Affiliations:** 1 Institute of Crop Science, Shandong Academy of Agricultural Sciences, Key Laboratory of Wheat Biology & Genetic Improvement on North Yellow & Huai River Valley, Ministry of Agriculture, National Engineering Laboratory for Wheat & Maize, Jinan 250100, China; zhangrongzhi1981@126.com (R.Z.); zsjhappy@163.com (S.Z.); lgy111@126.com (G.L.); 2 BGI Institute of Applied Agriculture, BGI-Agro, Shenzhen 518083, China; zhengfengya@genomics.cn; 3 Guangxi Botanical Garden of Medicinal Plants, Nanning 530023, China; weishugen2@163.com; 4 China Tobacco Gene Research Center, Zhengzhou Tobacco Research Institute of CNTC, Zhengzhou 450001, China

**Keywords:** disease resistance gene, miRNA regulation, CKRI, ETI, PTI, HIGS, SIGS

## Abstract

Biotic stresses do damage to the growth and development of plants, and yield losses for some crops. Confronted with microbial infections, plants have evolved multiple defense mechanisms, which play important roles in the never-ending molecular arms race of plant–pathogen interactions. The complicated defense systems include pathogen-associated molecular patterns (PAMP) triggered immunity (PTI), effector triggered immunity (ETI), and the exosome-mediated cross-kingdom RNA interference (CKRI) system. Furthermore, plants have evolved a classical regulation system mediated by miRNAs to regulate these defense genes. Most of the genes/small RNAs or their regulators that involve in the defense pathways can have very rapid evolutionary rates in the longitudinal and horizontal co-evolution with pathogens. According to these internal defense mechanisms, some strategies such as molecular switch for the disease resistance genes, host-induced gene silencing (HIGS), and the new generation of RNA-based fungicides, have been developed to control multiple plant diseases. These broadly applicable new strategies by transgene or spraying ds/sRNA may lead to reduced application of pesticides and improved crop yield.

## 1. Introduction

The arms race of plants and host-pathogens seems never to stop, and sometimes the race is very intense. During the evolutionary process, plants have had to evolve multiple immunity mechanisms to survive danger signals in extracellular and intracellular milieus. Plants are able to enhance disease resistance and increase the food security, as well as to balance the resource allocation between growth and development. The prevalent defense mechanisms are categorized into three defense layers: the preliminary defense, pathogen-associated molecular pattern (PAMP) triggered immunity (PTI) [1], the secondary defense, effector-triggered immunity (ETI) [2], and the additional defense, the exosome-mediated cross-kingdom RNA interference (CKRI) system [3]. 

It is well-known that PTI functions in basal defense. Using the cell surface-localized pattern recognition receptors (PRR), plants can detect the infection of invaders by recognizing the conserved microbe-associated or pathogen-associated molecular patterns (MAMPs or PAMPs) [1]. Plant PRRs are cell surface localized, and always are receptor-like kinases (RLKs) and receptor like proteins (RLPs). RLKs are comprised of extracellular domains, transmembrane domains, and intracellular kinase domains, which are required for transmitting the signals to the downstream defense responses, whereas RLPs are only comprised of the basic conformation without intracellular kinase domain. PTI with broad-spectrum defense is not sufficient to prevent most pathogens, and if plants have defect in PRRs, they often become more susceptible to microbes [4,5,6,7]. In turn, pathogens employ kinds of virulence effectors to overcome PTI and establish successful infection, termed effector-triggered immunity. Thus, ETI functions in the second defense of elicitor mediated defenses. 

Most of the genes involved in ETI pathway contain intracellular nucleotide-binding site and leucine-rich repeat domains (NBS-LRRs or NLRs), which are typically cytoplasmic receptor proteins. *NBS-LRR* genes can detect or recognize the polymorphic, strain-specific pathogen-secreted virulence effectors, and then transfer the signals to the downstream of defense genes. Thus, ETI-pathways belong to the species-specific disease resistance, and rapidly co-evolve with their pathogens. Plant species in eudicots and dicots have lots of *NB-LRR* genes. According to the N-terminal features and functions, the NB-LRR proteins in plants can be termed into two classes with the terminal Toll/interleukin-1receptor (TIR) or coiled-coil (CC)/resistance to powdery mildew8 (RPW8) domains [8,9,10]. The TIR, CC or RPW8 domains are crucial in signaling transmit in cellular targets for effector action or downstream signaling components [11]. Although the *NB-LRR* genes were demonstrated as the ancient and conserved genes in plants, their comparative genomic analyses have shown great structural diversity. For example, the CC domains are prevalent in eudicots and monocots, while the TIR domains are nearly absent in monocots [12]. Cross-kingdom RNA interference (CKRI) functions in the third layer, which protects plants by extracellular vesicles transport small RNAs or microRNAs (miRNAs) to microbial pathogens and then silence the virulence genes [3].

**Figure 1:**
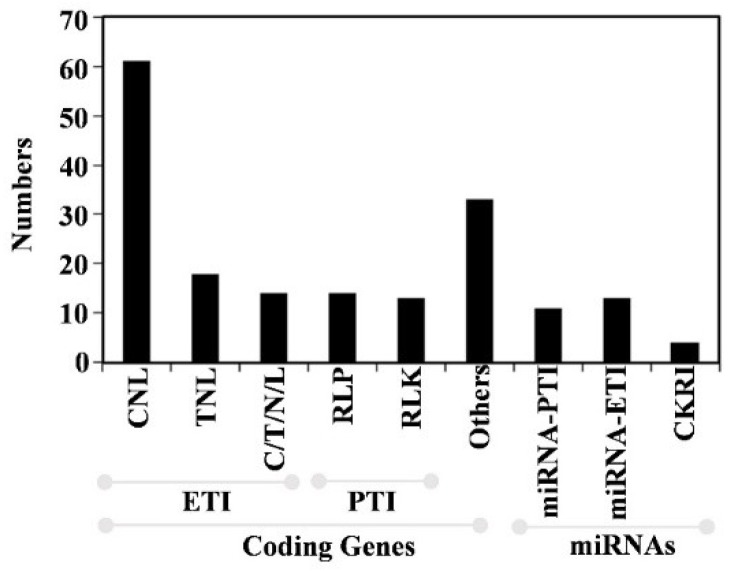
Categories of the genes/regulators in the three defense layers in plants. The data was downloaded from PRGdb database and the recent publications [20,21,22,23,24,25,26,27,28,29,30,31,32,33,34,35,36,37,38,39,40,41,42,43,44,45,46,47,48,49,50,51,52,53,54,55,56,57,58,59,60,61,62]. PTI: pathogen-associated molecular patterns (PAMP) triggered immunity; ETI: effector-triggered immunity; CRKI: cross-kingdom RNA interference.

As one kind of typically small non-coding RNAs, miRNAs function in post-transcriptional gene regulation. Small miRNAs play big roles in a variety of biological processes, such as development, hormone responses and stress adaptations [13,14,15,16]. In PTI and ETI pathways, microRNAs as the classical regulators in post-transcript or translation level regulate defense/defense-associated genes [17,18], which can balance the benefits and costs of their targets. Plants employ miRNAs as shields against the pathogen attacks. MiRNAs respond to virus, bacteria and fungi by negatively regulating of mRNAs, which mainly function in both PTI and ETI. Until now, totally 153 disease resistance genes from PRGdb database [19], which involved in the plant immunity to biotic stresses, were validated by experiments in wet labs. Of them, 62.09% (95 from 153) genes, 17.65% (27 from 153) genes, 20.26% (31 from 153) genes were classified as NBS-LRR families, RLP/RLK, and other kinds of genes, respectively (Figure 1). 

In regard to defense genes, studies have shown a number of genes/small RNAs linked to anti-pathogen immunity. Here, we mainly summarize the current knowledge of the defense genes and their evolution paths regulated by miRNAs in plants, and then discuss their potential applications in crop improvements in the last section. 

## 2. Three Layers of Defense Mechanisms to Biotic Stresses in Plants

### 2.1. The First Layer of Defense: Defense Genes in PTI

**Figure 2:**
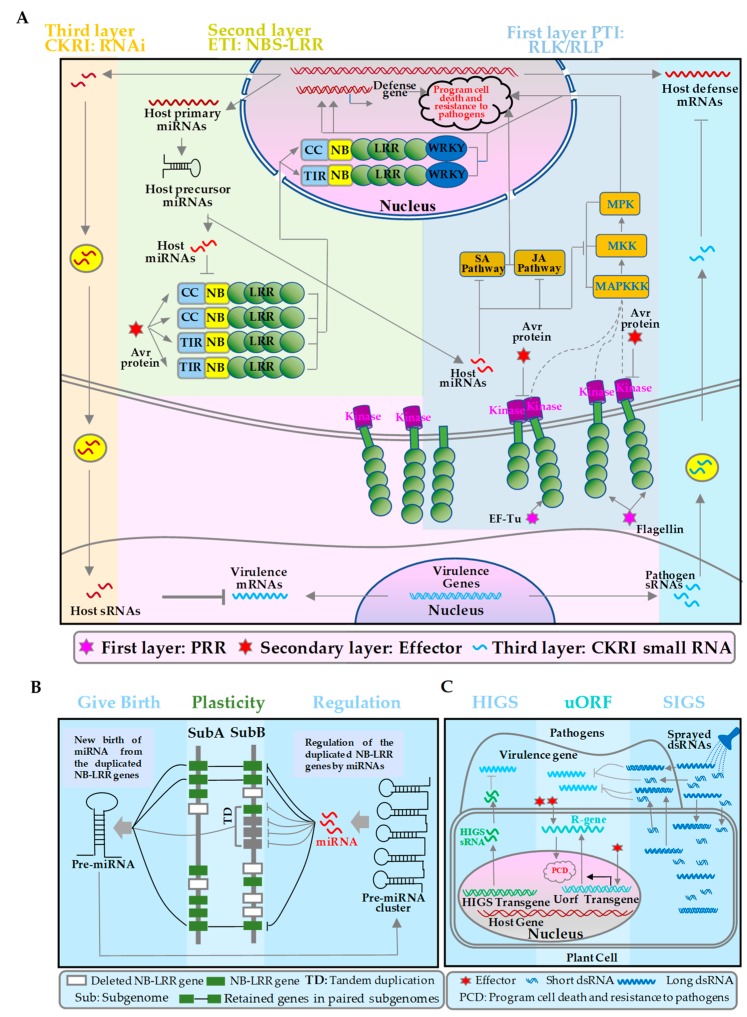
The interaction mechanisms of plants-pathogens from three interacted and miRNA regulation layers. (**A**) The three defensive layers in plants including the PTI, ETI, and cross-kingdom RNA interference (CKRI), and the three infection layers in pathogens including pattern recognition receptors (PRR), effector and CKRI. (**B**) The evolution of *NBS-LRR* genes and their regulator miRNAs. (**C**) The three strategies of defense to biotic stresses including uORF [83], host-induced gene silencing (HIGS) [84,85,86,87,88,89,90,91,92] and spray-induced gene silencing (SIGS) [3,93,94,95,96] in plants.

As one of the most important sensory protein groups, RLKs and RLPs in plants play crucial roles both in cell–cell and the plant–environment communications such as plant–pathogen interaction. In addition, RLKs and RLPs play fundamental roles in plant growth and development. Plants deploy a wide assay of RLKs and RLPs as the first layer of inducible defense to detect microbe- and host- derived molecular patterns (Figure 2A, the first layer) [63]. Numbers of RLKs/RLPs have been cloned in plants [64]. The best classical example is FLAGELLIN-SENSITIVE2 (FLS2), belonging to RLK family, which have been verified to response to Flagellin fragment flg22 of bacteria in *Arabidopsis* [65], grapevine [66], tobacco [67], rice [68] and tomato [69]. As a “molecular glue”, flg22 induces the activity of the heterodimerization complex FLS2-BAK1 (BRI1-ASSOCIATED RECEPTOR KINASE). In different plant species, FLS2 receptors display different affinities for the conserved part of flagellin from different bacteria, which possibly reflect the coevolution with specific-pathogens [66]. Except FLS2, EF-TU RECEPTOR (EFR), PEP 1 RECEPTOR (PEPR1), PEPR2, RLP23, RLP30 [70], the endogenous AtPep1 [71], NLPs [72], and SCFE1 [73], can also recognize bacterial EF-Tu, respectively. All of them are associated with the regulatory BAK1 that acts as a co-receptor for flg22/EF-Tu/AtPep1/nlp30/SCFE1 of pathogens and are crucial for signaling activation [74].

Long chitin oligomers as bivalent ligands, lead to the homodimerization of CHITIN ELICITOR RECEPTOR KINASE 1 (AtCERK1) and generate an active receptor complex in *Arabidopsis*, which directly trigger chitin-induced immune signaling [75]. The chitin perception system in rice is significantly different from the one in *Arabidopsis*. OsCERK1 dimmer does not bind chitin since the single LysM domain, while the dimer elicitor-binding LysM-RLP (OsCEBiP) can bind the chitin by ligand. The OsCERK1-chitin-OsCEBiP then forms a sandwich-type receptor dimerization for chitin oligomers [76].

There are a number of RLKs/RLPs involved in plant immunity, which have been well summarized by Tang et al [63]. After plant sensing of pathogen/microbe-associated molecular patterns, these pattern recognition receptors instantly trigger a number of downstream responses, such as the activation of mitogen-activated protein kinases (MAPKs) (Figure 2A, the first layer), which is one of the earliest signaling events [77]. By phosphorylation to transmit response signals, MAPKKK actives MKK, and then MKK actives MPK [78]. MAPK cascades is involved in multiple signaling defense responses, including the biosynthesis/signaling of plant stress/defense hormones, reactive oxygen species generation, stomatal closure, defense gene activation, phytoalexin biosynthesis, cell wall strengthening, and hypersensitive response (HR) cell death (Figure 2A, the first layer) [77]. The activation of MAPK cascades is essential for plant immunity. 

In addition, some transcription factors were found to regulate the defense-related genes that involved in signal transduction in rice. For example, a *bZIP* gene *OsBBI1* in rice, is a major transcription factor to regulate the resistance spectrum for diverse groups of *M. oryzae* by altering the first level of innate immunity in host plants [79]. WRKY13 as another major regulatory factor was identified to transfer signals from WRKY45 to downstream WRKY42 as functioning WRKY- type transcription factors (TFs) [80]. Following the SA-pathway-dependent disease response mechanism, WRKY13 shows correlation of the defense to *M. oryzae* and *Xoo* [81]. By activation of NPR1 protein, the SA pathway plays a crucial role in the systemic acquired resistance response mechanism (Figure 2A, the first layer) [82]. As a result, kinds of genes comprised of cellulase surface disease resistance genes and intracellular transcript factors could function in the complex PTI.

### 2.2. The Second Layer of Defense: The Defense Genes in ETI

In ETI pathway, plants have developed NBS-LRR proteins to recognize effectors and trigger the ETI response [2], which can cause programmed cell death together with the downstream of *WRKY* and lead to hypersensitive response (HR) (Figure 2A, the second layer) [97]. *NBS-LRRs* as an interesting class of disease resistance genes own a larger member in plants. In Table 1, about 1.19–3.48% of total coding genes were defined as *NBS-LRR* genes. Although *NB-LRR* genes are abundant in plants, only 93 genes are validated to play important roles in the innate immunity of plants up to now. Of the validated *NBS-LRR* genes, 65.59% (61 from 93) genes contain the CC domains, while only 19.35% (18 from 93) genes contain the TIR domains, and the others contain only one domain of either NBS, LRR, TIR, CC, or RPW8 (Figure 1). The verified disease resistance genes with CNL or TNL domains are listed in Table 2. For example, seven CNLs and seven TNLs in *Arabidopsis thaliana*, eleven CNLs in *Oryza sativa*, five CNLs and one TNL in *Solanum lycopersicium*, seven CNLs in *Triticum aestivum*, three CNLs in *Hodeum vulgare* had been exemplified by experiments. These defense genes in plants can confer the resistance to fungi, oomycetes, bacteria, viruses, nematodes, and insects. 

One type of plant disease can be prevented by several genes (Table 2). For example, the bacterial blight in *Arabidopsis* caused by *Pseudomonas syringae*/*Xanthomonas oryzae*, can be defended by RPM1 (CNL) [99], Rps2 (CNL) [100], RPS5 (CNL) [101], SSI4 (TNL) [102], and Rps4 (TNL) genes [103]. The downy mildew of cucurbits that caused by *Pseudoperonospora cubensis* (Oomycetes) in *Arabidopsis*, can be resisted by RPP13/RPP8 (CNL/CNL) [104], RPP1/RPP4 (TNL/TNL) [105,106], and RPP5 (TNL) [107,108]. In rice, the famous rice blast disease caused by *Magnaporthe grisea* or *Magnaporthe oryzae*, can be defended by 17 CNL type of disease resistance genes including Pi-ta/PIB [109], RGA5 [110], Pi36/Pi9/Pi2 [111,112,113], Piz-t/Pikm1-TS/Pikm2-TS/Pid3/Pi5-1/Pi5-2/Pit/Pikp-2 [113,114,115,116,117], Pia [118], Pi37 [119] and Rpr1 [120]. In barley, the powdery mildew caused by *Blumeria graminis*, can be resistant by CNL type of genes including MLA10 [121], MLA1 [122], and MLA13 [123]. In *Linum usitatissimum*, flax rust caused by *Melampsora lini* (Fungal), can be resistant by TNL type of genes including P2 [124], L6 [125], M [126], L [127], L1-L11 [128,129], P [129,130], and P1 [124]. One disease resistance gene can also confer plants resistant to several plant diseases (Table 3). For example, XA1 (CNL) [131] in rice, can defense to bacterial blight caused by bacterium of *Pseudomonas syringae* and *Xanthomonas oryzae*. Rx2 in *Solanum acaule*, can defense to potato virus X (Virus) and *Heterodera schachtii* (Nematode) [132].

The disease resistance genes were abundant in the wild resource. In *Triticeae* for example, the defense genes *Sr31* and *Sr50* [133] from cereal rye (*Secale cereale*), can confer the resistance to stem rust disease caused by *Puccinia graminis f. sp. tritici* (*Pgt*). *Sr35* gene from *Triticum monococcum* confers the resistance to Ug99 Stem Rust Race Group [134]. In addition, some non-*NBS-LRR* genes can also provide the defense to pathogens. For example, Stb6 in wheat can directly interacted with the effector AvrStb6 that produced by wheat pathogen *Zymoseptoria* *tritici* [135]. The X10 gene, which has four potential transmembrane helices in rice, can be induced by transcription activator–like (TAL) effector AvrXa10. The gene can confer disease resistance to rice bacterial blight by inducing programmed cell death in rice [136,137].

By introgression or transgene strategy, these defense genes confer the disease resistance in plants. For example, by overexpressing Pm3a/c/d/f/g in wheat, all tested transgenic lines showed the significantly more resistance than their respective non-transformed sister lines in field experiments [138]. The T0 and T1 transgenic lines with the *Sr50* gene were resistant to *Puccinia graminis f. sp. tritici* (*Pgt*), while lines without the transgene were susceptible [133]. 

### 2.3. The Third Layer of Defense: Cross-Kingdom/Organism RNA Interference

It had been demonstrated that plasmodesmata sRNAs can presumably move from cell to cell, and they systemically travel through vasculature [139]. Remarkably, sRNAs also move and induce their target gene silencing between interacted organisms and hosts. The phenomenon was defined as cross-kingdom/organism RNA interference (CKRI) [20,93,140,141,142]. Pathogens can deliver sRNAs into plants. It was recently discovered as a novel class of pathogen effectors (Figure 2A, the third layer). *Botrytis cinerea* can deliver small RNAs (Bc-sRNAs) to plant cells to silence host immunity genes [140]. Such small RNA effectors in *B. cinerea* are mostly produced by Dicer-like protein 1/2 (Bc-DCL1/2). In reverse, over-expressing sRNAs that target *Bc-DCL1* and *Bc-DCL2* in tomato and *Arabidopsis*, would silence *Bc-DCL* genes and inhibit fungal growth and pathogenicity. It exemplified bidirectional CKRI and sRNA trafficking between plants and fungi [93]. The easy traveling phenomenon suggests naturally occurring small RNAs might exchange each other across cross-kingdom/organism.

Conversely, hosts also can transfer naturally occurring small RNAs into pests or pathogens to attenuate their virulence (Figure 2A, the third layer). Recently, two reports have demonstrated that naturally occurring plant small RNAs might be delivered into pathogens to silence their target genes. In response to the infection of *Verticillium dahliae*, cotton plants increase the dose of miR159 and miR166 in expression level and then export both to the fungal hyphae for specific silencing. Two genes encoding an *isotrichodermin C-15 hydroxylase* and a *Ca*^2+^*-dependent cysteine protease*, were targeted by miR159 and miR166, respectively. Both of the target genes are essential for fungal virulence [20]. Another example is that host *Arabidopsis* cells by secreting exosome-like extracellular vesicles can also transfer small RNAs into fungal pathogen *Botrytis cinerea*. At the infection sites, these sRNA-containing vesicles accumulate and then are taken up by the fungal cells. Delivered host small RNAs induce the silence of fungal genes that is critical for pathogenicity. TAS1c-siR483 target two genes *BC1G_10728* and *BC1G_10508* from *B. cinerea*, and TAS2-siR453 targets *BC1T_08464*. All of the three genes involving in vesicle trafficking pathways are critical for pathogenicity [3]. Of them, *BC1G_10728* encodes a vacuolar protein sorting 51 and plays a crucial role in *Candida albicans* virulence [21]. Thus, *Arabidopsis* has adapted exosome-mediated CKRI mechanism as part of its immune responses during the evolutionary arms race with the pathogens [3]. 

Based on the above description, since only two miRNAs and two small RNAs in plants were identified to function in CKRI, data are inefficient to deduce their evolution among species. Thus, in the next section, we only discussed the evolution of disease resistance genes and their regulator miRNAs in PTI and ETI.

## 3. The Regulation of Disease Resistance Genes by Small RNAs

### 3.1. The First Layer of Defense Regulation: miRNAs Involved in the PTI Pathway

During pathogen infection, plant small RNAs play key roles in gene regulation level. According to the targets of miRNAs that how to respond to the pathogen infection, miRNAs were divided into active and repressed regulation in basal resistance (Figure 1A, Table 3). In the positive regulation, overexpression of miRNAs conferred the resistance to defense diseases in plants. For example, miR393 in *Arabidopsis*, was discovered to contribute to the antibacterial resistance by negatively targeting the transcripts of the *F-box auxin receptors TIR1* [22]. Repressing auxin signaling through miR393 overexpression increases bacterial resistance; conversely, augmenting auxin signaling through over-expressing a *TIR* enhances susceptibility to virulent *Pto DC3000*. miR444/*OsMADS* directly monitors *OsRDR1* transcription, and involves in the rice antiviral response [23]. Overexpression of miR444 enhanced rice resistance against rice stripe virus (*RSV*) infection by diminishes the repressive roles of *OsMADS23*, *OsMADS27a,* and *OsMADS57* and concomitant by the up-regulation of *OsRDR1* expression. Thus, miR444 can indirectly activate the *OsRDR1*-dependent antiviral RNA-silencing pathway. Over-expression of osa-miR171b conferred less susceptibility to rice stripe virus infection by regulating the target *OsSCL6*. *OsSCL6-IIa/b/c* was down-regulated or up-regulated in plants, where osa-miR171b was over-expressed or interfered [24].

In the negative regulation, overexpression their target genes could confer the resistance to pathogens in plants. miR169 suppresses the expression of *NFYA* in immunity against the infection of bacterial wilt *Ralstonia solanacearum* [25] and the blast fungus *Magnaporthe oryzae in Arabidopsis* and rice, respectively [26]. The transgenic lines of over-expressing miR169a, became hyper-susceptible to pathogens. MiR156 and miR395 regulate apple resistance to Leaf Spot Disease [27]. In apple, Md-miR156ab and Md-miR395 suppress *MdWRKYN1* and *MdWRKY26* expression, which decreases the expression of some pathogenesis-related genes, and results in susceptibility to *Alternaria alternaria f. sp. mali*. In *Arabidopsis*, miR396/*GRF* module mediates innate immunity against *P. cucumerina* infection without growth costs. Reduced activity of miR396 (MIM396 plants) was found to improve broad resistance to necrotrophic and hemibiotrophic fungal pathogens [28]. MiR319/*TCP* module involves in the rice blast disease. Increasing expression level of rice miR319 or decreasing expression level of its target *TCP21*, *LIPOXYGENASE2* (*LOX2*) and *LOX5* can facilitate rice ragged stunt virus (*RRSV*) infection [29], which caused the decreased endogenous jasmonic acid (JA) [30]. Inhibiting ath-miR773 activity accompanied with up-regulation of its target gene *METHYLTRANSFERASE 2* increased resistance to hemibiotrophic (*Fusarium oxysporum*, *Colletototrichum higginianum*) and necrotrophic (*Plectosphaerrella cucumerina*) fungal pathogens in *Arabidopsis* [31]. By regulating the transcription of *GhMKK6* gene in cotton, ghr-miR5272a involved in the immune response. Over-expressing ghr-miR5272a increased sensitivity to *Fusarium oxysporum* by decreasing the expression of *GhMKK6* and the followed disease-resistance genes, which lead a similar phenotype to *GhMKK6*-silenced cotton [32]. In addition, miRNAs could also be involved in the resistance to nematode invasion. For example, miR827 in *Arabidopsis* down-regulated the expression of *NITROGEN LIMITATION ADAPTATION* (*NLA*) gene. It suppressed the basal defense pathway by enhancing susceptibility to the cyst nematode *Heterodera schachtii* [33].

Except these miRNAs indirectly regulation the PTI pathway, a few of miRNAs were predicted to directly regulate the receptor-like genes. For example, when osa-miR159a.1 was repressed, the expression of *OsLRR-RLK2* was induced, which is responded to *Xanthomonas oryzae pv. Oryzae* in rice [31]. In future, some miRNAs regulation of pattern recognition receptors (PRR) genes may be validated by experiments.

### 3.2. The Second Layer of Defense Regulation: The Defense Signal Small RNAs in ETI

In addition to the basal defense, miRNAs are also involved in ETI pathway to directly and indirectly regulate the disease resistance genes (Figure 2A & Table 3). MiR393*, the complementary strand of miR393 within the sRNA duplex, by targeting a protein trafficking gene *Membrin 12* promote the secretion of antimicrobial PR proteins, which functions in ETI during infection of *Pseudomonas syringae pv. Tomato* in *Arabidopsis* [34]. The miR863-3p is induced by the bacterial pathogen *Pseudomonas syringae*. During early infection, miR863-3p silences two negative regulators of plant defense, namely *atypical receptor-like pseudokinase1* (*ARLPK1*) and *ARLPK2*, both of which trigger immunity through mRNA degradation. Later during infection, miR863-3p silences *SERRATE*, and positively regulates defense. And *SERRATE* is essential for miR863-3p accumulation by a negative feedback loop. Thus, miR863-3p targets both negative and positive regulators of immunity through two modes of action to fine-tune in the timing and amplitude of defense responses [35].

High expression of plant *NBS-LRR* defense genes is often lethal to plant cells, which is associated with the fitness costs. Thus, plants develop several mechanisms to regulate the transcript level of *NBS-LRR* genes. One of the key mechanism is the suppression of regulation network in microRNAs and *NBS-LRRs*, which may play a crucial role in plant-microbe interactions by sRNA silencing mechanism [18]. *NBS-LRR* genes confer defense against the pathogen infections in gene dosage dynamic expression level by multiple duplications and diversification, while miRNAs minimized the cost of gene copies by inhibiting their expression [36]. One miRNA can regulate dozens to hundreds of *NBS-LRRs* by targeting the similar motif sites [37], which make it more economical to balance the benefits and costs of these copies in genome. Until now, a few of miRNAs had been validated to be involved in the regulation of *NBS-LRR* genes. 

The regulation between miRNAs and *CC-NB-LRR* or *TIR-NB-LRR* gene classes was mostly characterized in eudicots. In most of the post-transcriptional regulation networks, the miRNA can trigger the 21-nt phased siRNA generation in *NB-LRR* transcripts, which were processed by RNA-dependent RNA polymerase 6 (RDR6) and DICER-LIKE 4 (DCL4) [38]. For example, in *Brassica* miR1885 were validated to induce by Turnip Mosaic Virus (*TuMV*) infection, which cleaved *TIR-NB-LRR* class genes [39]. In Tobacco, by cleaving *TIR-NB-LRR* immune receptors, both of nta-miR6020 and nta-miR6019 provide resistance to Tobacco mosaic virus (*TMV*) [40,41]. In tomato, sl-miR5300 and sl-miR482f controlled NB domain-containing proteins in mRNA stability and translation level, which involved in plant immunity [42]. In *Arabidopsis*, miR472 modulated the disease resistance genes mediated by RDR6 silencing pathway [43]. In *Medicago*, miR2109, miR482/miR2118 and miR1507 were found to influence *NB-LRR* gene family [37]. In legumes, miR482, miR1507, miR1510, and miR2109 suppressed *NB-LRR* gene class with CC or TIR domains, which were proposed to function in the regulation of defense response or host specificity during rhizobium colonization [38,44]. In addition, miR482/miR2118, miR946, miR950, miR951, miR1311, miR1312, miR3697, miR3701, and miR3709 were also mediated to generate phased siRNAs by targeting *NBS-LRR* gene class in Norway Spruce [45]. In monocots, miR2009 (also named miR9863 in miRBase) was first predicted in wheat to target the *Mla* alleles [46]. In barley, the miR9863 family was confirmed to trigger response to the *Mla* alleles [47].

## 4. The Evolution of Defense Genes

### 4.1. The Evolution of Defense Gene in PTI

In land plants, RLKs expanded extensively and fulfilled these diverse roles including perceive growth hormones, environmental/danger signals derived from pathogens [143]. In *Arabidopsis*, 44 RLK subgroups were defined, and leucine-rich repeat receptor-like kinases (LRR-RLK) belong to the largest receptor-like kinase family and are focused by researchers [144]. According to characters of unique basic gene structures and protein motif compositions, plant *LRR-RLKs* constitute 19 subfamilies, most of which were derived from the common ancestors in land plants. The proportions of *LRR-RLK* genes in Lycophytes and moss genome are 0.30% and 0.36%, respectively, while the proportions of *LRR-RLK* genes in angiosperms are 0.67–1.39% [145], which indicated the special expansion of defense genes in angiosperm genomes. *LRR-RLK* involved in the defense/resistance-related genes was less conserved than that involved in development. Defense-associated *LRR-RLKs* undergone many duplication events, and most of them were massively lineage-specific expansion mainly by tandem duplication [143,144]. These discoveries provide important resources for future functional research for these critical signaling genes in PTI.

### 4.2. The Evolution of Defense Gene in ETI

*NBS-LRR* genes as a class of ancient and conserved genes have been detected in gymnosperms, angiosperm plants and animals to ensure immunity [12,146,147]. However, comparative genomic analyses have demonstrated that *NBS-LRR* genes have a great structural diversity in plants and animals. For example, TIR domains were established in the ancestor plants conifers and mosses, and also in animals shared functionality regarding innate immunity [148,149,150]. TIR genes specially expanded in dicot genomes, but are absent or at least rare in monocot genomes [8,147,151,152,153]. For *NBS-LRR* genes, tandem duplication in genome is the major expansion mechanism in plants. More than 60% of *NBS-LRR* genes organized in a general pattern of clusters in plant genomes (Figure 2B) [98]. During whole genome duplication, biased deletions happened in the duplicated paralogous blocks with *NB-LRR* genes, and it could be possibly compensated by their local tandem duplication mechanism (Figure 2B). 

The miRNAs typically target highly duplicated *NBS-LRRs*, and families of heterogeneous *NBS-LRRs* were rarely targeted by miRNAs in *Brassicaceae* and *Poaceae* genomes [18]. miRNAs/*NBS-LRR*-genes interactions drove functional diploidization of structurally retained *NBS-LRR* genes duplicates by suppression regulation (Figure 2B) [98]. Evolutionary shuffling events such as diploidization and tandem duplication, leaded to copy number variations and presence absence variations in the synteny collapse of *NBS-LRR* genes [154,155,156,157]. In addition, the polymorphisms often exist in a population [158]. A contrasted conservation of *NBS-LRR* genes was observed with only 23.8% for monocots and 6.6% for dicots. Thus, *NBS-LRR* genes as one of the most plastic gene family in plants have less conservation such as synteny erosion or alternatively loss in plants compared with the other coding protein genes [98].

## 5. The Evolution of microRNAs in the Defense Pathway

### 5.1. The Evolution of miRNAs in PTI

In the PTI pathway, most of miRNAs were very conserved and directly/indirectly involve multiple biological processes in the development and abiotic/biotic stresses. All of the MiR169, miR171, miR393, miR395, and miR396 were ancient miRNAs present in both dicots and monocots [48]. miR444 was specific in monocots [49], whereas miR773 and miR5272 were lineage-specific in *Arabidopsis* and *Medicago*. The miRNAs conserved in plants mostly regulate the important transcript factors. These transcript factors tend to involve multiple biological processes. Take miR169 and miR396 for example, miR169/*NFYA* in *Arabidopsis* indirectly affected lateral root initiation [50], nitrogen-starvation [51], drought stress [52], and biotic stress [25,26]. In *Arabidopsis* roots, miR396/GRF regulates the switch between stem cells and transit-amplifying cells [53], which affects rice yield by shaping inflorescence architecture [54], and biotic stresses [28]. 

Both of the miRNA/target regulation and their function are very conserved in plants. MiR169/*NFYA* module influences the *Ralstonia solanacearum* pathogenicity in *Arabidopsis* [25] and the resistance to *M. oryzae* strains in rice [26]. In addition, these conserved miRNAs’ targets were expanded except for their classical miRNA/target model. For example, miRNA156 regulates of the SQUAMOSA-PROMOTER BINDING PROTEIN-LIKE (SPL) family involve in the timing of vegetative and reproductive phase change, which is highly conserved among phylogenetically distinct plant species [55]. miR395 by targeting a high-affinity sulphate transporter and three ATP sulfurylases involved in the sulfate homeostasis, is also conserved in plants [56,57]. Differently, both miR156 and miR395 regulate apple resistance to leaf spot disease by targeting *WRKY*. Thus, miRNAs involved in PTI pathway, are conserved in PTI defense pathway and in plant development such as miR393 *vs TIR* in auxin signal pathway [22] and miR319 *vs. TCL* in JA pathway [29]. Only few of miRNAs were reported to potentially regulating the *RLK*/*RLP* by osa-miR159a.1 [58], MiR5638 and miR1315 [59]. Genes involved in the PTI pathway were relatively conserved compared to these genes involved in ETI pathway. Thus, most of their regulator miRNAs were also conserved miRNAs or neofunctionalization of miRNAs in plants.

### 5.2. The Evolution of miRNAs in ETI

Although there are many miRNAs regulated *NB-LRR* genes, the conservation level of miRNAs is lower than the development associated miRNAs or PTI-associated miRNAs. In the eudicots and monocots, there is no conserved miRNAs targeting the *NB-LRR* genes. Lineage- or species-specific disease resistance-associated miRNAs were continually present and accompanies the continually varied pathogens. And some miRNAs with similar sequences had obvious functional diversity. miR482/miR2118 in eudicots mostly targeted *NB-LRR* genes, however, it only initiated the generation of 21-nt phased siRNAs in rice, and most of the target transcripts were noncoding sequences and specifically expressed in the rice stamen and the maize premeiotic and meiotic anther [60,61,62]. It clearly concluded that miR2118 initiated the phased siRNA in male reproductive organs. Therefore, a functional switch occurred in miR482/miR2118 between eudicots and dicots. Their expression level also varies in the lineage-related species. Tae-miR3117 was predicted to target the numbers of *NBS-LRRs* with higher expression in the tetraploid and hexaploid *Triticum* seedlings, while it had lower expression levels in *Aegilops tauschii* (not published data). And in rice, maize, and sorghum, miR3117 also displayed lower expression levels.

Diverse miRNAs, as negative transcriptional regulators, inhibit *NBS-LRRs* in plants. The highly duplicated *NBS-LRRs* were typically targeted by miRNAs (Figure 2B), while families of heterogeneous *NBS-LRR* genes were rarely regulated by miRNAs such as in *Poaceae* and *Brassicaceae* genomes. For example, some miRNAs also have a high duplication rate such as miR482/miR2118 in tandem duplication in genomes [60,61,62], which may enhance the expression dosage. 

Newly emerged miRNAs were periodically derived from duplicated/redundant *NBS-LRRs* from different gene families. And most of these new birth miRNAs target these *NBS-LRR* gene regions of conserved, encoded protein motif, which follow in the convergent evolution model (Figure 2B). The miRNAs may drive the rapid diploidization of these *NBS-LRR* genes in polyploid plants. These *NBS-LRR* associated miRNAs had a rapid diversity. The nucleotide diversity of the target site region in the wobble position of the codons drives the diversification of miRNAs. These characters of high duplication rate and rapid diversity were similar to their target genes. The co-evolutionary model between *NBS-LRRs* and miRNAs in plants makes the plants balance the costs and benefits of disease resistance [18].

## 6. The Strategies of Defense Pathogens in plants

### 6.1. The First Strategy: Utilize the Disease Resistance Genes by a Molecular Switch 

Up to now, a number of genes were exemplified to be involved in plant immunity defense. By over-expressing such defense genes can dramatically enhance disease resistance in plants, while is often associated with significant penalties to fitness and make the resulting products undesirable. Thus, it is difficult in agricultural applications. Recently, it has been developed a strategy to utilize these disease defense genes from the angle of plant genes or their regulators [83]. The strategy is to introduce immunity-inducible promoter and other two pathogen-responsive upstream open reading frames of the *TBF1* gene. It is called uORFsTBF1, which is a key immune regulator and its translation is transiently and rapidly induced upon pathogen challenge (Figure 2C, uORF). It has been demonstrated that inclusion of the *uORFsTBF1*-mediated translational control over the production of AtNPR1 in rice and an auto-activated immunity receptor snc1-1 in *Arabidopsis* did not reduce the plant fitness in the laboratory or in the field [83]. This strategy using a molecular switch enables us to engineer more broad-spectrum disease resistance genes with minimal adverse effects on plant growth and development in the agriculture application. 

### 6.2. The Second Strategy: Host-Induced Gene Silencing (HIGS)

Transgene-derived artificial sRNAs in plants can induce the target gene silencing in certain interacting insects [84,85], nematodes [86], fungi [87,88,89,90], oomycetes [91,92], and even plants–plants [141]. The phenomenon was called host-induced gene silencing (HIGS). The artificial sRNAs can travel from host plants to pathogens or pests and then function in trans (Figure 2C, HIGS). It had been well used in many plants in the decades. By plant RNAi suppressing a bollworm *P450 monooxygenase* gene of cotton impaired larval tolerance of gossypol [85]. In transgenic plants, by RNAi silencing of a conserved and essential root-knot nematode parasitism gene engineered broad root-knot resistance [86]. HIGS of nematode fitness and reproductive genes decreases fecundity of *Heterodera glycines Ichinohe*. Double-stranded RNA complementary to *cytochrome P450 lanosterol C14 alpha-demethylase-encoding* genes of *Fusarium* in *Arabidopsis* and barley contributes to strong resistance to *Fusarium* species [90]. HIGS to the *MAPKK* gene *PsFUZ7* in wheat enhance stable resistance to wheat stripe rust [159]. HIGS of an important pathogenicity factor *PsCPK1* in *Puccinia striiformis f. sp. tritici* conferred resistance of wheat to stripe rust [160]. By transgene-mediated cross-kingdome RNAi mechanism, HIGS by transgene is a good and effect strategy to improve the crop disease resistance in a broad spectrum.

### 6.3. The Third Strategy: Spray-Induced Gene Silencing (SIGS) 

The pathogens and pests are capable to take up the double RNAs or small RNAs from the plants or the environments [93]. Based on this and according to the mechanism of cross-kingdom/organism RNA interference, researchers have developed a strategy to control crop disease. It is spray-induced gene silencing (SIGS) that spraying dsRNAs and sRNAs on plant surfaces can target pathogen genes to repression pathogen virulence (Figure 2C, SIGS). For modern crop protection strategies, it is a natural blueprint. Evidences suggest that nematodes [94], insects [84] and fungi [95] could uptake up the environmental dsRNA or sRNAs. Directly spraying the dsRNAs that target the fungal *cytochrome P450 lanosterol C-14alpha-demethylases* of fungal gene can suppress fungal growth [95]. On barley leaves, spraying *CYP51*-targeting dsRNA at a concentration range of 1–20 ng/mL, inhibited growth of *Fusarium* species [3]. It has been demonstrated that spraying naked sRNAs and dsRNA on plants was successful to protect fruits and vegetables against pathogens. However, pesticide effect of the naked sRNAs and dsRNAs can only last 5–8 days. Mitter, et al. developed a method to load dsRNAs on designer, non-toxic, degradable, layered double hydroxide (LDH) clay nanosheets. This LDH made the dsRNA does not be wash off, and can be sustained released for 30 days [96]. This SIGS broadly application of new strategy may contribute to reduced use of chemical pesticides and lightening of selective pressure for resistant pathogens. The new-generation of RNA-based fungicides and pesticides are powerful, eco-friendly, which can be easily adapted to control multiple plant diseases simultaneously. 

## 7. Conclusions

Plants deployed PTI, ETI, and CKRI innate immune systems to arm race with different pathogen stresses. Pathogens developed more advanced effectors to defeat plant defense immunity. A number of genes have been exemplified to play important role between the host-pathogen interactions in plants. These signaling genes will be helpful to improve plant disease resistance against various pathogens. The sustainable and broaden spectrum resistance genes and their regulators such as miRNAs will be applied in developing crop varieties by introducing the molecular switch. From the cross-kingdom angle, the HIGS can also be used to crop breeding by transgenic approach, which can also confer the broaden spectrum resistance to hosts. The SIGS can also make plants yield the broaden spectrum resistance by spraying the designed dsRNAs/sRNA. Further function studies in plants will dissect more and more defense genes and hopefully unravel the intricate defense regulation network. More and more molecular technologies will be invented and adapted to help develop the eco-friendly disease-resistance cultivars.

## Figures and Tables

**Table 1:** Disease resistance genes and their regulator miRNAs in plants [98]. Mbp, million base pair; Nb, number; R-gene, disease resistance genes.

Species	Nb Chr.	Size (Mbp)	Nb Gene	R-Genes			
				Nb R-Genes	(%) ^1^	Nb MiRNA Targets	(%) ^2^
**Monocots**							
*Oryza sativa* (rice)	12	372	41,046	1196	2.91	144	12.04
*Sorghum bicolor*	10	659	34,008	625	1.84	109	17.44
*Zea mays* (maize)	10	2365	32,540	673	2.07	0	0
*Brachypodium distachyon*	5	271	25,504	537	2.11	149	27.75
**Eudicots**							
*Arabidopsis thaliana*	5	119	33,198	503	1.52	81	16.1
*Populus trichocarpa*	19	294	30,260	446	1.47	382	85.65
Carica papaya	9	234	19,205	228	1.19	0	0
*Glycine max*	20	949	46,164	1171	2.54	290	24.77
*Malusxdomestica* (apple)	17	742	58,979	2052	3.48	256	12.48

^1^ the percentage of the R-genes from the total coding genes; ^2^ percentage of the miRNA target genes from the R-genes.

**Table 2:** The validated disease resistance genes and their pathogens in plants. The data were derived from PRGDB database.

Plant Species	Disease	Pathogens	Avirus Genes	Types of Pathogens	Genes	Types	GenBank Locus
*Arabidopsis thaliana*	White rust of crucifers	*Albugo candida*		Oomycetes	RAC1	TNL	AY522496
	Cucumber Mosaic Virus	*Cucumber mosaic virus*		Virus	RCY1	CNL	AB087829
	Bacterial Blight	*Pseudomonas syringae/Xanthomonas oryzae*	avrRpm1; avrRpt2; avrPphB; N; avrRps4	Bacterium	RPM1; Rps2; RPS5; SSI4; Rps4	CNL; CNL; CNL; TNL; TNL	NM_111584; NM_118742; NM_101094; AY179750; NM_123893
	Downy mildew of cucurbits	*Pseudoperonospora cubensis*		Oomycetes	RPP13/RPP8; RPP1/RPP4; RPP5	CNL/CNL; TNL/TNL; TNL	NM_114520/NM_123713; NM_114316/NM_117790; NM_117798
	Bacterial wilt of potato	*Ralstonia solanacearum*		Bacterium	RRS1	TNL	NM_001085246
	Turnip crinkle virus	*Turnip crinkle virus*		Virus	HRT	CNL	NM_128190
*Aegilops tauschii*	Cereal cyst nematode	*Heterodera avenae*		Nematode	Cre1	CNL	AY124651
*Capsicum chacoense*	Bacterial spot of tomato	*Xanthomonas campestris*	AvrBs2	Bacterium	Bs2	CNL	AF202179
*Capsicum chinense*	Pepper mild mottle virus	*Pepper mild mottle virus*		Virus	L3	CNL	BAJ33559
*Cucumis melo*	Fusarium Wilt	*Fusarium oxysporum*		Fungal	FOM-2	CNL	DQ287965
	Melon aphid disease	*Aphis gossypii*		insect	VAT	CNL	AGH33848
	zucchini yellow mosaic virus	*zucchini yellow mosaic virus*		Virus	FOM1	TNL	AIU36098
*Glycine max*	Soybean mosaic virus	*soybean mosaic virus*		Virus	KR1	TNL	AF327903
*Helianthus annuus*	Downy mildew of sunflower	*Plasmopara halstedii*		Oomycetes	Pl8	CNL	AY490793
*Hordeum vulgare*	Powdery mildew (barley)	*Blumeria graminis*		Fungal	MLA10/MLA1/MLA13	CNL	AY266445; GU245961; AF523683
*Lactuca sativa*	Downy mildew of lettuce	*Bremia lactucae*	Avr3	Oomycetes	Dm3 (RGC2B)	CNL	AH007213
*Linum usitatissimum*	Flax rust	*Melampsora lini*		Fungal	P2/L6/M; L,L1-L11; P,P1-4	TNL; TNL; TNL	AF310960/U27081/U73916; AAD25974/AAK28806
*Nicotiana glutinosa*	Tobacco Mosaic Virus	*Tobacco mosaic virus*		Virus	N	TNL	U15605
*Oryza sativa*	Rice blast disease	*Magnaporthe grisea*	Avr-Pita	Fungal	Pi-ta/PIB	CNL	AY196754
	Bacterial Blight	*Pseudomonas syringae/Xanthomonas oryzae*		Bacterium	XA1	CNL	AB002266
	Rice blast disease	*Magnaporthe oryzae*		Fungal	RGA5	CNL	EU883792
*Oryza sativa Indica Group*	Rice blast disease	*Magnaporthe grisea*		Fungal	Pi36/Pi9/Pi2	CNL	DQ900896/DQ285630/DQ352453
*Oryza sativa Japonica Group*	Rice blast disease	*Magnaporthe grisea*		Fungal	Piz-t/Pikm1-TS/Pikm2-TS/Pid3/Pi5-1/Pi5-2/Pit/Pikp-2	CNL	DQ352040/AB462324/AB462325/FJ773286/EU869185/EU869186/AB379816/HM035360
	Rice blast disease	*Magnaporthe oryzae*		Fungal	Pia; Pi37; Rpr1	CNL; CNL; CNL	AB604626; DQ923494; AC119670
*Solanum acaule*	Latent mosaic of potato/Beet cyst nematode	*Potato virus X/Heterodera schachtii*		Virus/Nematode	Rx2	CNL	AJ249448
*Solanum bulbocastanum*	Late Blight of tomato	*Phytophthora infestans*		Oomycete	Rpi-blb1/Rpi-blb2; RB	CNL; CNL	AY336128; DQ122125; AY426259
*Solanum demissum*	Late Blight of tomato	*Phytophthora infestans*		Oomycete	R1	CNL	AF447489
*Solanum lycopersicum*	Bacterial spot of tomato	*Xanthomonas campestris*	Hax4/AvrBs4	Bacterium	Bs4	TNL	AY438027
	Yellow potato cyst nematode	*Yellow potato cyst nematode*		Nematode	Hero	CNL	AJ457052
	root-knot nematode	*Meloidogyne incognita*		Nematode	Mi1.2	CNL	AF039682
	Tomato Spotted Wilt	*Tomato spotted wilt virus*		Virus	Sw-5	CNL	AY007366
	Tobacco Mosaic Virus	*Tobacco mosaic virus*	MP	Virus	Tm-2a/Tm-2	CNL	F536201/AF536200
*Solanum pimpinellifolium*	Bacterial Speck of tomato	*Pseudomonas syringae*	AvrPto/AvrPtoB	Bacterium	Prf	CNL	AF220602
	Late blight	*Phytophthora infestans*		Oomycete	Ph-3	CNL	KJ563933
*Solanum tuberosum*	Yellow potato cyst nematode	*Globodera*		Nematode	Gpa2	CNL	AF195939
	Late Blight of potato	*Phytophthora infestans*		Nematode	Gro1.4	TNL	AY196151
	Latent mosaic of potato/Beet cyst nematode	*Potato virus X/Heterodera schachtii*		Virus	Rx	CNL	AJ011801
*Solanum tuberosum subsp andigena*	Potato virus Y	*Potato virus Y*		Virus	RY-1	TNL	AJ300266
*Triticum aestivum*	Brown wheat rust of potato	*Puccinia triticina*		Fungal	Lr10/Lr21/Lr1	CNL	AY270157/FJ876280/EF439840
	powdery mildew	*Blumeria graminis f. sp. Tritici*		Fungal	Pm3	CNL	AY325736
	stem rust	*Puccinia graminis f. sp. Tritici*		Fungal	Sr33	CNL	KF031303
	Nematode disease	*Heterodera avenae*		Nematode	Cre3	CNL	AF052641
	Yellow rust	*Puccinia striiformis Westend. f.sp. Tritici*		Fungal	Yr10	CNL	AF149114
*Triticum monococcum subsp. Monococcum*	stem rust	*Puccinia graminis f. sp. Tritici*		Fungal	Sr35	CNL	AGP75918
*Zea mays*	Common rust of maize	*Puccinia sorghi*		Fungal	Rp1-D	CNL	AF107293

**Table 3:** List of regulators involved in the immunity response to pathogens in plants.

Plant miRNAs	Immunity Response	Targets in Plants or Pathogens	Positive (+)/Negative (−) Regulator	Pathogens	
				Classification	Pathogen/Plant
miR393	PTI	F-box auxin receptors	Positive	Bacteria	*Pseudomonas syringae*/*Arabidopsis*
miR160a	PTI	auxin response factors16	Positive	Bacteria	*Pseudomonas syringae*/*Arabidopsis*
miR319	PTI	TCP21	Negative	Virus	*Rice ragged stunt virus* (RRSV)/RICE
miR773	PTI	METHYLTRANSFERASE 2	Negative	Bacteria; Fungul	*Pseudomonas syringae*/*Arabidopsis*; *Plectosphaerella cucumerina/Arabidopsis*
miR169	PTI	NFYA	Negative	Bacteria; Fungul	*Magnaporthe oryzae*/RICE
miR396	PTI	GRF	Negative	Fungul	*Plectosphaerella cucumerina*/*Arabidopsis*
miR156	PTI	MdWRKYN1	Negative	Fungul	*Alternaria alternaria*/APPLE
miR395	PTI	MdWRKY26	Negative	Fungul	*Alternaria alternaria*/APPLE
miR5272	PTI	MKK6	Negative	Fungul	*Fusarium oxysporum*/COTTON
MIR398	PTI	CSD2	Negative	Bacteria	*Pseudomonas syringae*/*Arabidopsis*
miR164	PTI	NAC	Negative	Fungul	*Magnaporthe oryzae*/RICE
miR393*	ETI	MEMB12 (Membrin 12)	Positive	Bacteria	*Pseudomonas syringae*/*Arabidopsis*
miR444	ETI	MADS	Positive	Virus	*Rice stripe virus* (RSV)/RICE
miR171	ETI	OsSCL6-Iia/b/c	Positive	Virus	*Rice stripe virus* (RSV)/RICE
miR863-3p	ETI	ARLPK1&ARLPK2	Positive	Bacteria	*Pseudomonas syringae*/*Arabidopsis*
miR863-3p	ETI	SERRATE	Negative	Bacteria	*Pseudomonas syringae*/*Arabidopsis*
MIR9863	ETI	*NBS-LRR*	Negative	Fungul	*Blumeria graminis*/Barley
MIR482	ETI	*NBS-LRR*	Negative	Fungul	*Fusarium oxysporum*/Tomato
MIR5300	ETI	*NBS-LRR*	Negative	Fungul	*Fusarium oxysporum*/Tomato
miR1510	ETI	*NBS-LRR*	Negative	Fungul	*Phytophthora sojae*/Soybean
miR6019	ETI	*NBS-LRR*	Negative	Virus	*Tobacco mosaic virus*/*Tobacco*
miR6020	ETI	*NBS-LRR*	Negative	Virus	*Tobacco mosaic virus*/*Tobacco*
miR1885	ETI	*NBS-LRR*	Negative	Virus	*Turnip mosaic virus*/*Brassica*
miR472	ETI	*NBS-LRR*	Negative	Bacteria	*Pseudomonas syringae*/*Arabidopsis*
miR166	CKRI	Clp-1 ^1^	Positive	Fungul	*Verticillium dahliae/Cotton*
miR159	CKRI	HiC-15 ^1^	Positive	Fungul	*Verticillium dahliae/Cotton*
TAS1c-siR483	CKRI	Bc-Vps51&Bc-DCTN1 ^1^	Positive	Fungul	*Botrytis cinerea /Arabidopsis*
TAS2-siR453	CKRI	BC1T_08464 ^1^	Positive	Fungul	*Botrytis cinerea /Arabidopsis*

CKRI: Cross-kingdom RNA interference; ^1^ Target gene from pathogen.
